# microDuMIP: target-enrichment technique for microarray-based duplex molecular inversion probes

**DOI:** 10.1093/nar/gku1188

**Published:** 2014-11-20

**Authors:** Jung-Ki Yoon, Jinwoo Ahn, Han Sang Kim, Soo Min Han, Hoon Jang, Min Goo Lee, Ji Hyun Lee, Duhee Bang

**Affiliations:** 1 College of Medicine, Seoul National University, Seoul 110-799, Korea; 2 Department of Chemistry, Yonsei University, Seoul 120-752, Korea; 3 Department of Pharmacology, Pharmacogenomic Research Center for Membrane Transporters, Brain Korea 21 PLUS Project for Medical Sciences, Severance Biomedical Science Institute, Yonsei University College of Medicine, Seoul 120-752, Korea; 4 Yonsei Cancer Center, Division of Medical Oncology, Department of Internal Medicine, Yonsei University College of Medicine, Seoul 120-752, Korea; 5 Department of Oral Biology, Yonsei University College of Dentistry, Seoul 120-752, Korea

## Abstract

Molecular inversion probe (MIP)-based capture is a scalable and effective target-enrichment technology that can use synthetic single-stranded oligonucleotides as probes. Unlike the straightforward use of synthetic oligonucleotides for low-throughput target capture, high-throughput MIP capture has required laborious protocols to generate thousands of single-stranded probes from DNA microarray because of multiple enzymatic steps, gel purifications and extensive PCR amplifications. Here, we developed a simple and efficient microarray-based MIP preparation protocol using only one enzyme with double-stranded probes and improved target capture yields by designing probes with overlapping targets and unique barcodes. To test our strategy, we produced 11 510 microarray-based duplex MIPs (microDuMIPs) and captured 3554 exons of 228 genes in a HapMap genomic DNA sample (NA12878). Under our protocol, capture performance and precision of calling were compatible to conventional MIP capture methods, yet overlapping targets and unique barcodes allowed us to precisely genotype with as little as 50 ng of input genomic DNA without library preparation. microDuMIP method is simpler and cheaper, allowing broader applications and accurate target sequencing with a scalable number of targets.

## INTRODUCTION

The advent of massively parallel sequencing, so-called next-generation sequencing (NGS), has enabled researchers to sequence the human genome in cost- and time-efficient manner (1). To capture genomic regions of interest for NGS, a variety of target enrichment strategies have been used such as microdroplet polymerase chain reaction (PCR) (2), solid-phase capture (3) and solution-phase capture (4,5) methods. Molecular inversion probe (MIP) capture, one of solution-phase captures, is using single-stranded oligonucleotides consisting of a backbone sequence flanked by annealing arms, which complement the genomic sequences next to the target (4). Compared with other target-enrichment methods, MIP-based methods have several advantages. They show high target specificity, require less genomic DNA (gDNA), have scalable number of targets and do not require preprocessing steps such as DNA fragmentation prior to capture experiments (6–8). After the MIP assay was developed for multiplexed genotyping of single nucleotide polymorphisms (SNPs) (9–12), it has become widely applied to detect copy number variations (CNVs) (8), RNA editing (13), methylation profiles (14,15), germline mutations (16,17), somatic mutations (18) and genotypes in duplicated genes (19).

**Figure 1.**
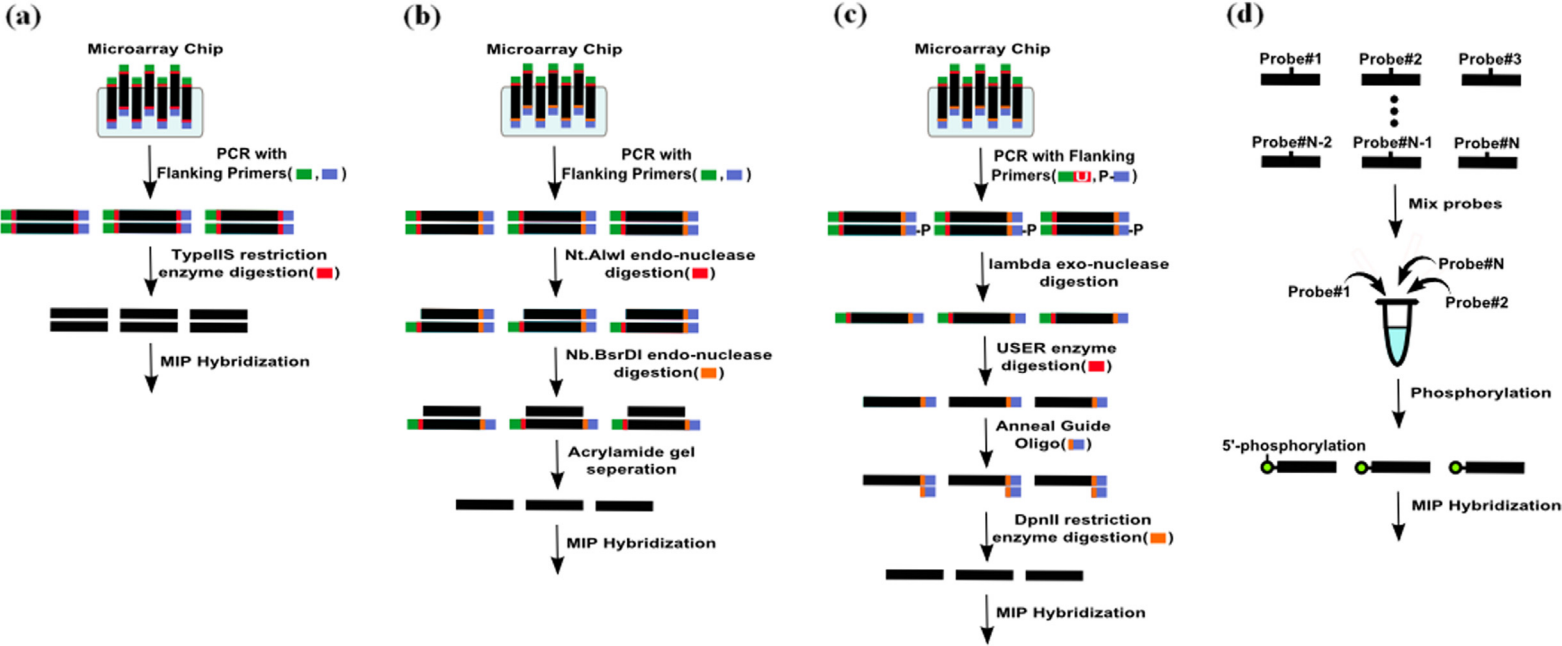
Comparison of MIP preparation protocols. (**a**) Microarray-based probe preparation method used in this study. (**b** and **c**) Conventional microarray-based methods. (**d**) Column-based probe preparation method.

To produce thousands of MIPs that capture a large number of targets simultaneously, oligonucleotides are typically synthesized and released from custom-designed microarrays (10). However, preparing single-stranded oligonucleotides from microarrays requires a number of laborious steps, such as endo- or exo-nuclease digestion and gel separation, which result in losing the vast majority of MIP products (Figure 1b and c) (20). Since the final yield is low, extensive PCR amplification is indispensable, which generates unexpected products and uneven probe quantities. Alternatively, column-based MIP synthesis generates probes one by one, producing a pure probe set (Figure 1d). As the cost of column-based oligonucleotide synthesis has dropped, column-based MIP capture has become a widely used capture method for hundreds to thousands of targets with (hundreds to) thousands of samples. Also, recent advancements, such as rebalancing, subpooling and molecular tagging, have improved the capture uniformity and sensitivity for detecting low frequent variants (16–18). Despite this, microarray-based synthesis has significant advantages in terms of cost producing probes at the 10k-scale.

MIPs have been developed in the form of single-stranded oligonucleotides since it has been considered as the complementary nucleotides may hinder the capture process. Interestingly, a recent study showed that double-stranded MIP captures both strands of the target, improving genotyping sensitivity and target coverage of hard-to-capture regions (21). However, this method requires thousands of individual PCR amplifications and has the limitations at scalability as column-based probe production. To our knowledge, there are no reports of microarray-based synthesis of double-stranded MIPs that can produce thousands of probes simultaneously at high quantities.

Filtering out PCR duplicates is used to exclude PCR amplification bias, and is a routine process in whole exome or genome sequencing data analysis. However, this step could not been applied to MIP experiments, because the original MIP-based capture products and their PCR duplicates align to the same positions on the genome. Recently, to mark sequences derived from common DNA fragments, single molecular tagging techniques have been successfully adapted to MIP capture method. Inserting these tags on column-based MIPs can improve the genotype sensitivity for low-frequency variants (18). We applied this single molecule tagging approach to microarray-based duplex MIP to remove PCR duplicates.

**Figure 2.**
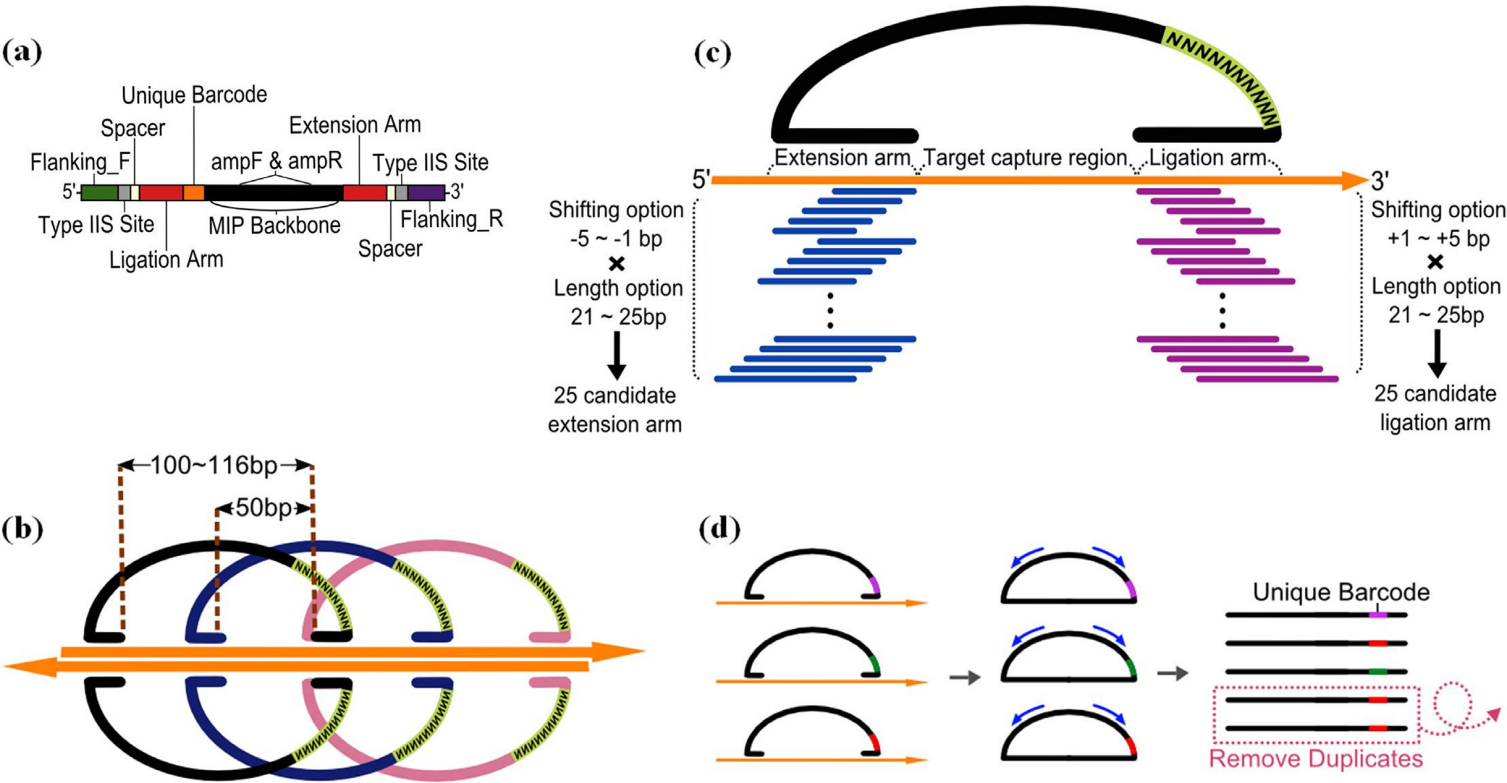
Major features of microDuMIP. (**a**) The composition of microDuMIP. (**b**) 2x Tiling design for both microDuMIP strands. (**c**) Selection of arm sequences from 25 candidates. (**d**) Use of unique barcodes to remove PCR duplicates.

Integrating recent advances in MIPs and using microarrays to produce probes in a scalable manner, we report an improved MIP capture method, called microarray-based duplex molecular inversion probe (microDuMIP), which has three main features: (i) double-stranded probes simplify the probe preparation, (ii) the probes contain overlapping targets to minimize missing regions by having neighboring probes and (iii) each probe contains a ‘unique barcode’ to remove PCR duplicates, thereby improving the genotyping accuracy (Figure 2). With this advancement, the microarray-based MIP approach can be not only simpler, more convenient and cheaper, but also is accurate with a scalable number of targets.

## MATERIALS AND METHODS

### Design of microDuMIP

microDuMIPs were designed with annealing arm sequences (extension/ligation arm), a unique barcode and post-capture PCR primer annealing regions (AmpF, AmpR) (Figure 2a). We initially designed 100 bp target regions with 50 bp of overlap (2x tiling density) (Figure 2b). For the extension and ligation arm sequences that capture target regions, we considered 25 candidates for each arm with five arm lengths ranging from 21 to 25 bases and five shifts of the start/end of the annealing region ranging from 0 to 4 bases (Figure 2c). We removed candidates meeting the exclusion criteria: EarI restriction site, redundancy on the genome (see below), homopolymers (more than eight consecutive identical bases) or extreme GC contents (<10% or >90%). The best of the remaining candidates was selected based on melting temperature and GC contents. Melting temperature was calculated by the nearest neighbor method with empirical thermodynamic parameters (22). Both the extension and ligation arms were designed to have melting temperatures as close to 60°C and GC contents as close to 50% as possible. All arm lengths and annealing temperatures were based on the Watson strand. Because of the asymmetry of the EarI enzyme site, microDuMIPs for targets on the Crick strand had slightly different arm lengths and annealing temperatures. To avoid each MIP capturing multiple genomic regions, each arm sequence was aligned to human genome by using BLAT (23) with the following parameters: for gfServer -stepSize = 5, for gfClient -minScore = 0 -minIdentity = 0. These parameters have been suggested by the developer to mimic results obtained by web-based Blat in the UCSC Genome Browser. If the arm sequence candidate is perfectly aligned to more than one genomic position, we considered that the candidate has redundancy on the genome and excluded it.

With the different arm lengths and shifting positions, gap-filling lengths ranged from 100 to 116 mer. We limited the gap-filling length to this because the product size is one factor that can cause PCR bias (24,25), and we expected that this approach minimized post-capture PCR amplification bias. Arm sequences were added to both ends of the microDuMIP, and EarI enzyme sites were added next to each annealing arm with ‘spacers’. The spacers were added to prevent EarI from cutting arm sequences. A unique barcode, 10 random nucleotides, was added next to the extension arm (Figure 2d). Each microDuMIP contained one of ∼10^10^ ( = 11,510×4^10^) unique barcodes to distinguish unique circularized capture products from PCR duplicates. Primer sequences (AmpF, AmpR) were attached for post-capture amplification.

### Microarray-based synthesis of MIP

Using a microchip-based oligonucleotide synthesis, 11 510 oligonucleotides were synthesized, and this oligonucleotide pool contained 6.93 μg of single-stranded oligonucleotide in 80 μl (86.61 ng/μl) (CustomArray^®^ Inc.). A total of 0.5 μl of oligonucleotide pool, 10 μl of KAPA HiFi polymerase (KAPA BIOSYSTEMS), 8 μl of dH_2_O with 1 μl of each forward and reverse primer (Supplementary Table S1) were used for probe amplification with the following PCR conditions: 95°C for 3 min; 15 cycles of 30 s at 95°C, 30 s at 60°C and 30 s at 72°C; and 10 min at 72°C. PCR-amplified samples were agarose-gel loaded and the correct bands were purified with a QIAGEN gel-extraction kit. We cleaved the flanking sequences with 1.5 μl of EarI (NEB^®^ Inc) and 3.5 μl of NEB buffer (NEB^®^ Inc) per 45 μl of probe template for 8 h at 37°C (Figure 1a). The products were purified with a QIAGEN gel-extraction kit and stored at 4°C.

### Target capture sequencing using microDuMIP

We diluted probes to make each probe species a 45 pM probe-mixture pool. Different amounts (50, 100, 200, 500 ng) of gDNA from HapMap sample NA12878 (Coriell) and microDuMIP-mixture corresponding to each condition were serially mixed with 1.5 μl of Ampligase buffer (Epicentre^®^), and dH_2_O was used to make a total volume of 15 μl. Hybridization started with 5 min at 95°C followed by denaturing for 5 min at 95°C, ramped at 0.1°C/s, and incubation for desired time (12–48 h) at 60°C. Then, 2 U of AmpliTaq^®^ DNA polymerase (Life Technologies), 4 U of Ampligase DNA ligase (Epicentre^®^), 10x dNTPs (NEB^®^ Inc), 0.2 μl of Ampligase buffer (Epicentre^®^) were added and the mixtures were incubated for 24 h at 60°C. Next, 0.5 μl of Exonuclease I (NEB^®^ Inc) and 0.5 μl of Exonuclease III (NEB^®^ Inc) were used to remove linear DNA fragments at 37°C, and a 5-min incubation at 95°C followed for deactivation. The post-amplification reaction used 1 μl of hybridized template, 10 μl of QIAGEN Multiplex PCR Master Mix (QIAGEN), 8 μl of dH_2_O with 1 μl each of Capture_AmpF and Capture_AmpR primers (Supplementary Table S1). The PCR conditions were 15 min at 95°C; 24–28 cycles of 30 s at 95°C, 30 s at 60°C, and 30 s at 72°C; and 30 min at 72°C. By using reverse transcriptase-polymerase chain reaction (RT-PCR), we determined the minimal number of PCR cycles needed to the plateau phase of RT-PCR curve. The PCR products were checked on an agarose gel, and the desired size bands were purified with a QIAGEN gel-extraction kit.

PCR-amplified target sites were phosphorylated with 3 μl of T4 polynucleotide kinase (NEB^®^ Inc) and incubated at 37°C for 8 h before PCR purification was performed. The adapter ligation step was followed for Illumina sequencing. The adapter sequences are listed in Supplementary Table S1. Thirty microliters of phosphorylated samples, 3 μl of T4 DNA ligase (Enzymatics), 3 μl of T4 DNA ligase buffer and 2 μl of each Illumina adapter primer were used and incubated at 20°C. Next, 20 μl of adapter-ligated template, 10 μl of KAPA HiFi polymerase (KAPA BIOSYSTEMS), 16 μl of dH_2_O and 2 μl of each forward and reverse adapter flanking primer, Flanking_F and Flanking_R (Supplementary Table S1), were added for the amplification and selection PCR. The PCR conditions were 3 min at 95°C; eight cycles of 30 s at 95°C, 30 s at 60°C, and 60 s at 72°C; and 10 min at 72°C. The PCR products were checked on an agarose gel, and the desired size bands were purified with a QIAGEN gel-extraction kit. The PCR products were sequenced using a HiSeq2500 (Illumina) platform according to manufacturer's instructions (Supplementary Figure S1).

### Bioinformatics and data analysis for target capture sequencing

Raw data were separated according to sample-specific barcodes (Supplementary Table S1). The extension and ligation arm sequences of microDuMIPs were detected in pair-end reads, and only gap-filled sequences (100∼116 mer), that is, target sequences, were identified. Novoalign (V2.07.18; www.novocraft.com) aligned target sequences to the human reference genome (hg19) with default parameters. For more precise calling SNVs around indels, GATK RealignTargetCreater and IndelRealigner (version 2.7.2) were used (26). PCR duplicates were removed as described below. SNVs were called by GATK UnifiedGenotyper (27) for each sample separately with the parameters -genotype_likelihoods_model BOTH -stand_call_conf 50 -stand_emit_conf 30. SNVs in the microDuMIP target regions with a depth of least 8x were used for further analysis. Format converting, sorting and indexing were performed by Samtools (28) (Supplementary Figure S2). Statistical package R and *effects* module applying a linear model were used for multivariate analysis to identify factors affecting sequencing product yield.

### Removal of PCR duplicates using unique barcode sequences

Read pairs with the same ‘unique barcode’ and microDuMIP arm sequences were considered PCR duplicates. The best pair, defined as the pair having the highest sum of base quality scores ≥15, was stored and duplicates were removed. Building consensus reads from PCR duplicates (18) is a more robust method than discarding reads with low mean base qualities, especially when sequencing reads are not enough or capture with low-quality DNA. Nonetheless, we used discarding strategy first, since this method is simple and same approach to Picard MarkDuplicates (http://picard.sourceforge.net), one of the most widely used modules in genome study.

### Validation of SNPs using the whole exome and Sanger sequencing

We compared SNPs called by microDuMIP capture with consensus SNPs from the Bottle consortium. To calculate the genotype concordance, positive predictive value and sensitivity of microDuMIP capture, SNPs from the Bottleneck consortium (29) were assumed to be correct. Also, Sanger validation of SNPs, especially those called from microDuMIP capture only, was performed. Primers for selected SNPs were designed with melting temperatures ranging from 57 to 62°C by Primer3web (version 4.0.0; http://primer3.wi.mit.edu) (30) (Supplementary Table S2). PCR conditions were as follows: 3 min at 95°C; 30 cycles of 30 s at 95°C, 30 s at 60°C, 30 s at 72°C; and 10 min at 72°C. PCR products were purified and sent for Sanger sequencing (Macrogen) and sequenced data were analyzed with SeqMan (DNASTAR).

### In-house programs and sequencing data

The scripts used for microDuMIP design, arm sequence identification and PCR duplicate removal are available upon request to D.B. (duheebang@yonsei.ac.kr). The deindexed and aligned data are available in the National Center for Biotechnology Information Sequence Read Archive (NCBI-SRA) as project SRP043024.

## RESULTS

### Targeted capture sequencing using microDuMIP

**Figure 3.**
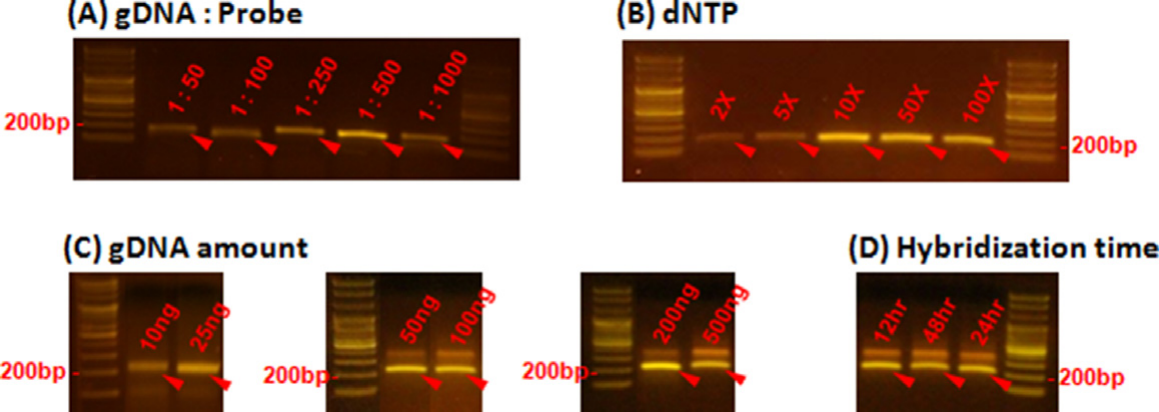
Comparison of capture efficiencies under different conditions. The effects of varying (**A**) the gDNA:probe ratio, and (**B**) the amount of dNTPs are shown. Band intensity (∼200 bp, red arrows) is proportional to the amount of captured product on each of the capture parameters, because the number of PCR cycles was held constant at 26 cycles. The amount of captured products was saturated at 1:500 gDNA:probe ratio and 10x dNTPs. (**C**) and (**D**) Captured products were detected around 200 bp for all conditions, and only products in these bands were separated and used for further analysis.

To validate our approach, we designed and synthesized microDuMIPs to capture 3554 exons including all protein coding regions of 228 cancer-related genes with splicing junctions (∼0.7 Mbp) (31) (Figure 2; Supplementary Table S3). To determine the most appropriate arm sequence, the melting temperatures and GC contents of 25 candidates were compared (Figure 2c; Supplementary Figure S3). A total of 11 510 target regions (11 510 of 12 406, 92.8%) were designed, and the other 896 regions were filtered out because all arm candidates contained a restriction enzyme site (323, 2.6%), were redundant in the human genome (493, 4.0%), were homopolymers (47, 0.4%) or contained extreme GC contents (44, 0.4%). Owing to the overlapping target strategy, 96.0% coverage of the initial target sequences was achieved (Supplementary Table S4). We adjusted several reaction conditions to optimize capture performance. Because double-stranded probes may act differently from conventional single-stranded probes, we performed capture experiments with NA12878 sample while altering the following parameters: gDNA:probe ratio, amount of dNTPs, amount of gDNA and hybridization time (Figure 3). With our preparation protocol producing high quantity of probes, microDuMIPs can be added at a higher ratio; however, the captured products were saturated at 1:500 gDNA:probe ratio (Figure 3A). Also, the amount of dNTPs was optimized at 10x, which was similar to conventional MIP experiments (12) (Figure 3B). To optimize the amount of gDNA (50, 100, 200, 500 ng) and hybridization time (12, 24, 48 h), we performed capture experiments and subsequent analysis via massively-parallel sequencing under 1:500 gDNA:probe ratio and 10x dNTPs (Figure 3C and D; Supplementary Table S5).

### Removing PCR duplicates and quantifying capture products by unique barcode

Removing PCR duplicates is essential to improve genotype calling accuracy. Picard MarkDuplicates (http://picard.sourceforge.net), one of the most popular codes for this step, identifies PCR duplicates based on the aligned positions. However, it cannot be applied to conventional MIP capture data, because the original capture product and its duplicates have the same target sequences and aligned positions. We integrated the ‘smMIP’ strategy (18) to microDuMIP method to track independent capture events, and inserted a ‘unique barcode,’ consisting of 10 random nucleotides on the microDuMIP backbone (Figure 2d). The number of possible barcodes and arm sequence combinations is ∼10^10^ ( = 11 510×4^10^) and 2.7–6.6 million combinations were observed for each condition (Table 1). Also, 749 847 distinct barcodes were detected at least once, indicating that 10-mer barcodes were not saturated yet (4^10^ = 1 048 576). These numbers are enough to preclude the possibility of any two microDuMIPs coincidently having same barcode; therefore, PCR duplicates can be distinguished. We selected a capture product from the pool of PCR duplicate products based on mean base qualities, which is analogous to Picard MarkDuplicates (Figure 2d), and used these unique reads for further analysis. In filtering the PCR duplicates, on average, 71% of reads were duplicates. As expected, less gDNA produced fewer capture products and more PCR duplicates. Among the 11 million aligned reads from the capture experiment with 50 ng gDNA, only 2.9 million (12.5%) reads were unique and the others were discarded (Table 1). Although the genotypes and allelic fractions of SNPs were not significantly different before and after removing PCR duplicates (Supplementary Figure S4), removal of PCR duplicates improved the precision of SNP calling, especially when using a small amount of gDNA (Supplementary Table S6).

**Table 1.** Capture specificity and percentage of usable reads according to capture conditions

Conditions		Raw	Mapped to genome		Mapped to TR		Unique reads	
gDNA (ng)	Hybridization time (h)	Reads (M)	Reads (M)	Percent (%) of reads	Reads (M)	Capture specificity (%)	Reads (M)	Percent (%) of reads
50	24	33.9	23.1	68.2	22.8	98.7	2.9	12.5
100	24	18.5	12.7	68.7	12.6	98.8	2.9	22.9
200	24	14.6	10.3	70.1	10.2	98.9	3.3	32.9
500	12	11.5	8.0	69.5	7.9	99.0	2.7	34.7
500	24	19.3	13.0	67.5	12.9	99.0	5.8	44.6
500^a^	24^a^	18.9	12.9	67.9	12.7	99.1	5.3	41.9
500	48	18.9	10.8	57.3	10.7	99.1	6.6	61.0

For all conditions, approximately 99% of reads were aligned to their own target positions. From 2.7 to 6.6 millions of unique reads, those reads with distinct barcodes and arm sequence combinations, were detected after the removal process of PCR duplicates, and were used for further analysis. A 1:500 gDNA:probe ratio and 10x dNTPs were used for all conditions.

TR: targeted regions.

^a^Replications for 500 ng of gDNA with 24 h of hybridization time.

### Capture performances

**Figure 4.**
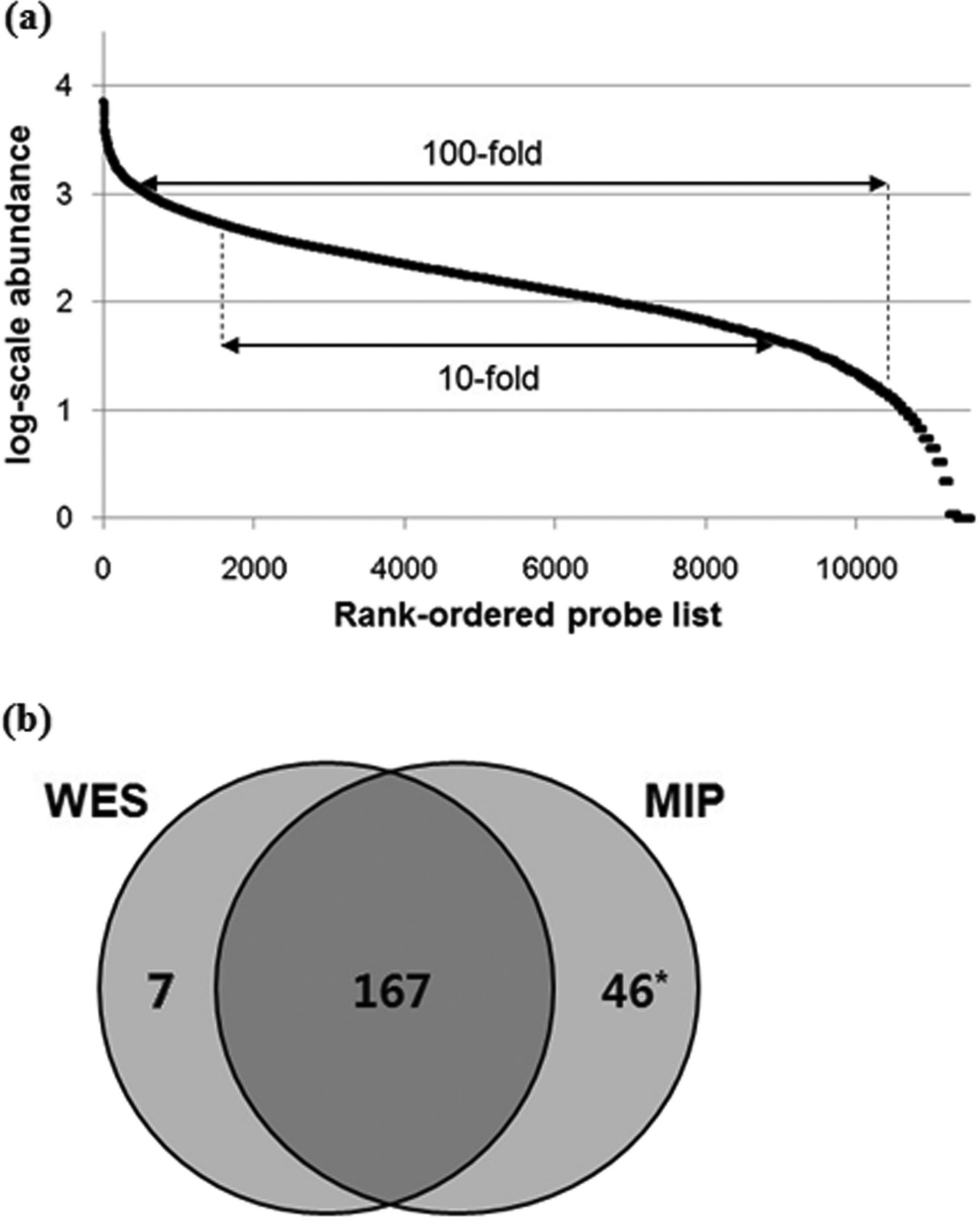
Capture uniformity and precision of the microDuMIP method. (**a**) Capture uniformity of the microDuMIP capture method. The graph compares the rank-ordered probe list (x-axis) versus the log-scale captured read abundance (y-axis); 98% of probes were detected at least once. A total of 61.3% of the capture regions were within a 10-fold range and 88.2% within a 100-fold range, which are comparable to results obtained with conventional capture methods, 57–58%, and 88–93%, respectively. (**b**) Comparison of microDuMIP captured SNPs with consensus SNPs. The number of SNPs in targeted regions of NA12878. WES: whole exome sequencing (Bottle consortium data); MIP: molecular inversion probe capture (500 ng of gDNA, 24-h hybridization time) *Of 46 SNPs called by MIP capture only, 37 loci were validated by Sanger sequencing, and 33 of 37 SNPs (89%) were determined to be true SNPs.

The performance of microDuMIP capture was evaluated with different amounts of input gDNA (50–500 ng, 24-h hybridization, 10x dNTPs, 1:500 gDNA:probe) (Table 1). More gDNA led to better capture specificity, but the capture specificities of all conditions were adequate (minimum 98.7% for 50 ng, maximum 99.1% for 500 ng) and comparable to those of conventional MIP capture, 99% capture specificity (11). Sensitivity and capture uniformity were also comparable to that of the conventional approach (Figure 4a). A total of 97.7% of capture regions were detectable, 61.3% within a 10-fold, and 88.2% within a 100-fold range, which are similar to the conventional capture results, 93–98, 57–58 and 88–93%, respectively (11,12). The performance of microDuMIP capture with different hybridization times (12, 24, 48 h, 500 ng of gDNA, 10x dNTPs, 1:500 gDNA:probe) was also evaluated (Table 1). As expected, a longer hybridization time resulted in much better capture specificity, but all conditions resulted in >99.0% capture specificity. We showed that capture uniformities for different hybridization times were similar because all conditions were already at maximal uniformity.

### Reproducibility

To test the reproducibility of microDuMIP capture, two independent capture experiments were performed under the same conditions (500 ng of gDNA, 24-h hybridization time, 10x dNTPs, 1:500 gDNA:probe). The allele fractions and depth of SNP loci between the two replicates were highly correlated (Pearson's correlation coefficient *R* = 0.964 and 0.947, respectively) (Supplementary Figure S5). When comparing between the 213 SNPs called by replicate 1 and the 214 SNPs called by replicate 2, we observed 100% genotype concordance between 208 out of 219 SNPs (95%) called by both replicates. Eleven SNPs were detected in either replicate 1 or 2, but the depth of coverage at those loci was less than 30x (mean 9.6x), suggesting those differences were mainly due to low coverage.

### Precision of SNP calling

Next, we explored the precision of SNP calling. The integrated, high-confidence SNPs of NA12878 called by the Bottle Consortium (29) were used as a reference standard. The precision of microDuMIP capture was acceptable; 99.5% genotype concordance and 96.5% sensitivity on average (Figure 4b). Moreover, microDuMIP capture sequencing identified missing SNPs from the integrated high-confidence set from the Bottle Consortium (whole exome sequencing data). We validated the missing SNP by Sanger sequencing (Supplementary Table S2); even with 50 ng of gDNA, 30 of 34 (88%) SNPs detected by microDuMIP capture only were true SNPs according to Sanger validation. The best reaction conditions for precision of SNP calling were 500 ng gDNA and 48-h hybridization time, but all conditions had a similar level of calling precision (Supplementary Table S6). Capture with 50 ng of gDNA had comparable genotype concordance (100%) and sensitivity (92%) to capture with 500 ng of gDNA.

### Quantification of circularized MIP products

**Figure 5.**
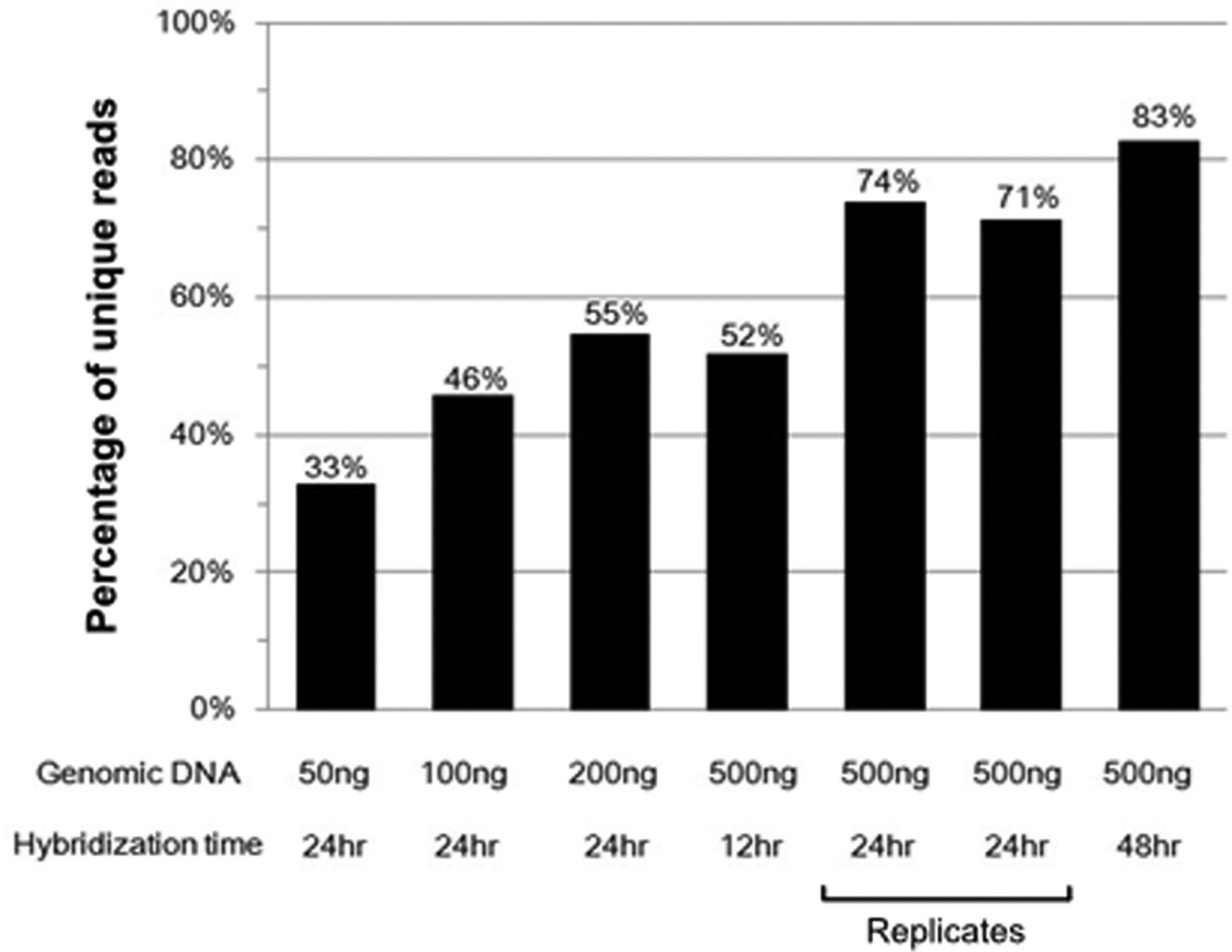
Relative quantity of circularized MIP products. The y-axis shows the ‘percentage of unique reads’, with unique reads defined as those having distinct barcodes and arm sequence combinations. Since the number of unique reads increases as total number of reads increases, in order to normalize it, we randomly selected 2 million reads for each condition, counted the number of unique reads and calculated the percentage of unique reads. As the amount of gDNA or hybridization time increased, more unique reads were detected, indicating that more circularized product was obtained. A 1:500 gDNA:probe ratio and 10x dNTPs were used for all conditions.

Since the unique reads defined as those having distinct barcodes and arm sequence combinations are proportion to the amount of circularized MIP products, we could quantify the amount of circularized products under different gDNA amounts and hybridization times (Figure 5). As expected, more gDNA and longer hybridization time produced more circularized MIP products. The relative quantities of two conditions (200 ng of gDNA with a 24-h hybridization and 500 ng of gDNA with a 12-h hybridization) were similar. Specific gDNA and hybridization conditions might be traded off depending on specific needs.

Factors that affected the amount of circularized MIP products were also investigated (Supplementary Figure S6). Probe melting temperature other than 60°C in the extension and ligation arms decreased the amount of MIP products (*P* = 0.00047 and *P* = 0.019, respectively). GC contents other than 50% in extension and ligation arms also decreased the amount of MIP products (*P* = 0.03 and *P* = 1.62×10^−5^, respectively). The lengths of the extension and ligation arms correlated with the amount of MIP products (*P* = 7.23×10^−7^ and *P* < 2×10^−16^, respectively). Precursor amounts were also found to influence the amount of MIP products (*P* < 2×10^−16^). Since we have observed uneven distribution of precursor microDuMIP (Supplementary Figure S7), we expect that utilizing more uniform microDuMIPs would be critically important to improve capture uniformity in microDuMIP capture. We checked how SNPs on MIP arms affected the amount of MIP products. Of 11 510 microDuMIPs, 218 (1.9%) probes had at least one SNP of NA12878 on either extension or ligation arm sequences, and those probes had lower amount of MIP products (*P* = 0.0013), indicating that SNPs on arm sequence also affected the capture performance of microDuMIP (Supplementary Figure S6h).

## DISCUSSION

Microarray-based nucleotide synthesis is a cheap and useful approach for producing a large number of probes simultaneously. O'Roak *et al*. (16) reported that 44 genes in 192 samples were successfully captured by 2069 MIPs produced using a column-based method; the total cost was ${\$ 0.33}$ per gene per sample, which is similar in scale to the estimated cost of capturing 228 genes in 60 samples with 11 510 microDuMIPs: ${\$ 0.22}$ per gene per sample (Supplementary Table S7). Although the price of column-based probe synthesis has dropped (${\$ 7.1}$ per probe (18)), the initial price for a 12 000 probe set produced by microarray is approximately 85-fold cheaper (${\$ 1000}$ versus ${\$ 85\ 000}$), resulting in increasing the total cost of column-based MIPs for capturing hundreds of genes. This cost estimate for microarray is based on the cost of synthesis reagents and a blank microchip, and it assumes the availability of a microchip synthesis machine from CustomArray, Inc. Although the price would be up to ${\$ 2500}$ without the synthesis machine, microarray-based probe preparation still has a cost advantage when producing probes at 10k-scale and when capturing hundreds of genes (Supplementary Table S7).

Conventional MIP has been designed with single-stranded DNA fragments to avoid self-aggregation. Thus, when starting with microarray oligonucleotides, previous studies for the generation of single-stranded MIPs required multiple enzymatic and purification steps, resulting in low probe yields (10,15). In contrast, we produced a high quantity of probes in straightforward manner with a typeIIS restriction enzyme digestion, and directly used the double-stranded DNA in capture reactions (Figure 1a). Despite previous concerns about self-aggregation, capture performance and precision of SNP calling with our double-stranded MIPs were comparable to conventional single-stranded MIP capture.

In a recent study (16), it was reported that hybridization could be performed together with polymerization and ligation, thus simplifying the protocol over conventional MIP capture methods. In double-stranded microDuMIP, which might provide for an increased likelihood of probe self-ligation, we indeed observed self-ligation products when all reagents were added during the denaturation step (Supplementary Figure S8). However, these bands were not dominant, were clearly distinguishable and were weaker when a smaller amount of probes were used. Therefore, adding gap filling reagents in advance can simplify this experimental step. In addition to this, we used gel purifications to compare capture performance using various experimental conditions and to visually check the products and byproducts in each step. Since we have established and confirmed the protocol, bead purification, which is a more efficient and easier method, would be recommended for analyzing samples.

The target regions of most conventional MIPs do not overlap because previous research assumed that sharing targets may hinder capture by other MIPs and that using minimal number of probes helps to reduce the cost of column-based probe synthesis. Owing to our protocol at low cost, we could allow the target regions of microDuMIP to overlap, and most genomic coordinates were covered by more than one microDuMIP. Even if one MIP failed to capture a target region, it could be captured by other adjacent MIPs (Figure 2b). To verify whether our overlapping target approach blocked capture, we divided 11 510 probes into two subsets that do not share target regions: 5740 ‘even’ probes and 5770 ‘odd’ probes. We synthesized the two subsets separately and performed the capture experiment in two tubes under the same conditions (50–500 ng gDNA for each tube, 24-h hybridization time, 10x dNTPs, 1:500 gDNA:probe). The capture performance and precision of SNP calling (Supplementary Table S8) in the two tubes with non-overlapping probes was not superior to a single tube experiment. For example, 50 ng of gDNA for two tubes used 100 ng of gDNA total, but the results were not better than those obtained with 50 ng of gDNA in a single tube. This indicates that shared targets may not hinder the capture process in microDuMIP. Considering the number of unique reads, this might result from the low efficiency of MIP capture. Only a small fraction of gDNA template is used during capture process, and there is minimal chance for overlapping MIPs to target the same template molecule.

When analyzing NGS reads, removing PCR duplicates is important to reduce amplification bias and obtain precise genotypes. We excluded PCR duplicates by adding a unique barcode (Figure 2d), which improved the precision of SNP calling with a small amount of input gDNA. As the depth of MIP capture increased, the percentage of duplicate reads also increased (Supplementary Figure S9), indicating that removing PCR duplicates is more important for ultra-deep re-sequencing or capture with little gDNA, such as FFPE samples. Although PCR duplicates had a small effect on our experiment, microDuMIP capture using unique barcodes can minimize bias from PCR steps and allow high-quality genotype calls. In MIP-based experiments, the depth distribution of variants can vary greatly, so this barcode-based approach has a distinct benefit.

In this study, in order to investigate the effects of PCR duplication, we sequenced at a much higher depth than what is usually required. For example, approximately 20% of targeted bases were sequenced at more than 1000x coverage in the capture experiment with 500 ng of gDNA and 24-h hybridization time, yielding a duplicate rate of 45%. According to the simulation, we estimate that sequencing 50, 25 and 12.5% of raw reads would increase the percentage of unique reads 57, 72 and 83%, respectively. Even with 5 millions raw reads (25%), the amount of reads sequenced 3554 exons of approximately 60 samples together on one Illumina Hiseq2500 lane, 96.9% of target regions were covered at least 1x and a 94.8% sensitivity of SNP calling was achieved. In addition to this, stopping the reaction prior to reaching the plateau of RT-PCR curve may reduce PCR duplicates as well.

Deng *et al*. (24) reported that the target length, GC content of the ligation arm and length of the ligation arm were correlated with MIP capture efficiency. In the current experiment, we fixed the gap-filling length to minimize post-capture PCR bias and to control the target length effect on the efficiency of microDuMIP capture. Statistical analysis showed that the abundance of circularized MIP products in microDuMIP capture, determined by the number of unique reads, is correlated with GC contents of not only the ligation arm but also the extension arm (Supplementary Figure S6). Because we used double-stranded probes, the ligation arm on the Watson strand may act as an extension arm on the Crick stand and vice versa. Therefore, either arm with an optimal melting temperature can act as a ligation arm, resulting in higher depths. Also, the amount of circularized MIP product is strongly correlated with the number of precursors in microDuMIP capture; therefore, rebalancing of probe quantities (24) might be critical to improve capture performance of microDuMIP further. Common SNPs on annealing arm sequences may affect annealing temperatures, and this feature was considered in the recent MIP design program (32). However, in our experimental setup, we did not consider the common SNP effect for probe design. We observed that SNPs of NA12878 on arm sequences were also contributing for capture performance of microDuMIP (Supplementary Figure S6h). Utilizing a probe design program (32) that is considering common SNP effects on arm sequence would be beneficial in the future.

Because we used the restriction enzyme EarI to produce microDuMIPs from the amplified precursors, no probe could contain an EarI recognition site in its arm sequences. Because of this limitation, which does not exist for a column-based approach, we failed to design probes for 323 (2.6%) target regions. However, the number of failed regions was not significant, and an overlapping target strategy rescued some of those regions. More importantly, this limitation could be overcome by adding one mismatched base to the EarI recognition site of the arm sequences.

In summary, our study showed that microarray-based MIP preparation can be simplified by using only on type IIS restriction enzyme with double-stranded MIPs, and that overlapping targets may not interfere with capture processes. Also, PCR duplicates can be excluded in MIP capture, and removing PCR duplicates may improve SNP calling precision for capture with little amount of gDNA. Even with our simplified protocol, the capture performance and precision of SNP calling of microDuMIP capture were comparable to those of conventional MIP capture. The capture performance and precision with as little as 50 ng of gDNA were almost the same as those with more gDNA, leading to the potential use of microDuMIP method for genotyping FFPE samples or circulating-tumor DNA. Although our method will additionally require iterative design to reach near completeness and uniformity, we believe that microDuMIP capture can be further improved by rebalancing probe uniformity.

## SUPPLEMENTARY DATA

Supplementary Data are available at NAR Online.

SUPPLEMENTARY DATA
