# Supplementary material for: microDuMIP: target-enrichment technique for microarray-based duplex molecular inversion probes
Source: Nucleic Acids Res. 2014 Nov 20;43(5):e28. doi: 10.1093/nar/gku1188 (PMC4357688; doi:10.1093/nar/gku1188)
Supplement: SUPPLEMENTARY DATA [file supp_43_5_e28__index.html]

microDuMIP: target-enrichment technique for microarray-based duplex molecular inversion probes — microDuMIP: target-enrichment technique for microarray-based duplex molecular inversion probes — SUPPLEMENTARY DATA 

# microDuMIP: target-enrichment technique for microarray-based duplex molecular inversion probes

## SUPPLEMENTARY DATA

**Files in this Data Supplement:**

- SUPPLEMENTARY DATA
- SUPPLEMENTARY DATA
