# Supplementary material for: microDuMIP: target-enrichment technique for microarray-based duplex molecular inversion probes
Source: Nucleic Acids Res. 2014 Nov 20;43(5):e28. doi: 10.1093/nar/gku1188 (PMC4357688; doi:10.1093/nar/gku1188)
Supplement: SUPPLEMENTARY DATA [file supp_gku1188_nar-01689-met-g-2014-File007.doc]

**Supplementary Figures**

Supplementary Figure S1. MIP experiment flow scheme


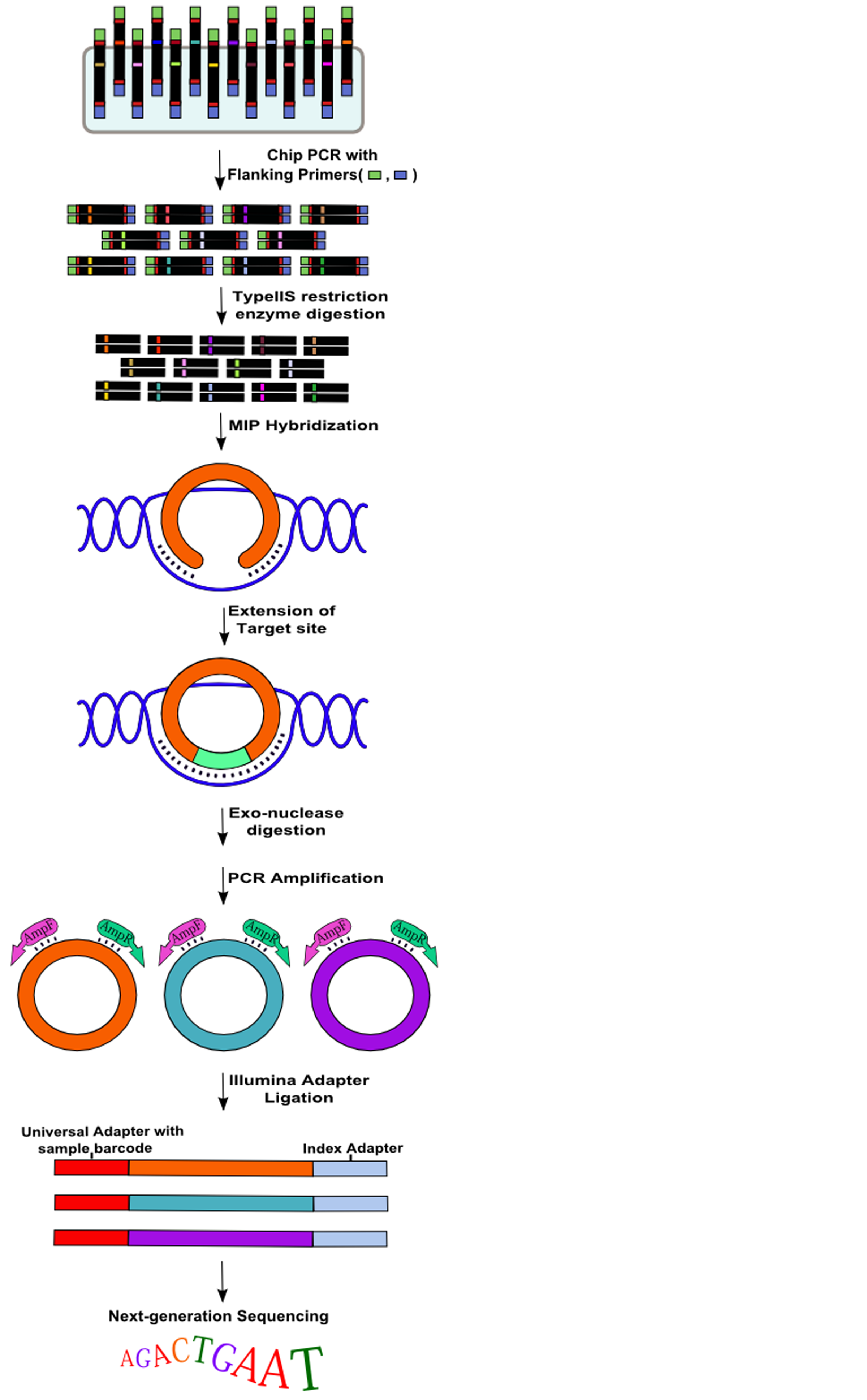


Supplementary Figure S2. Flowchart of microDuMIP capture analysis


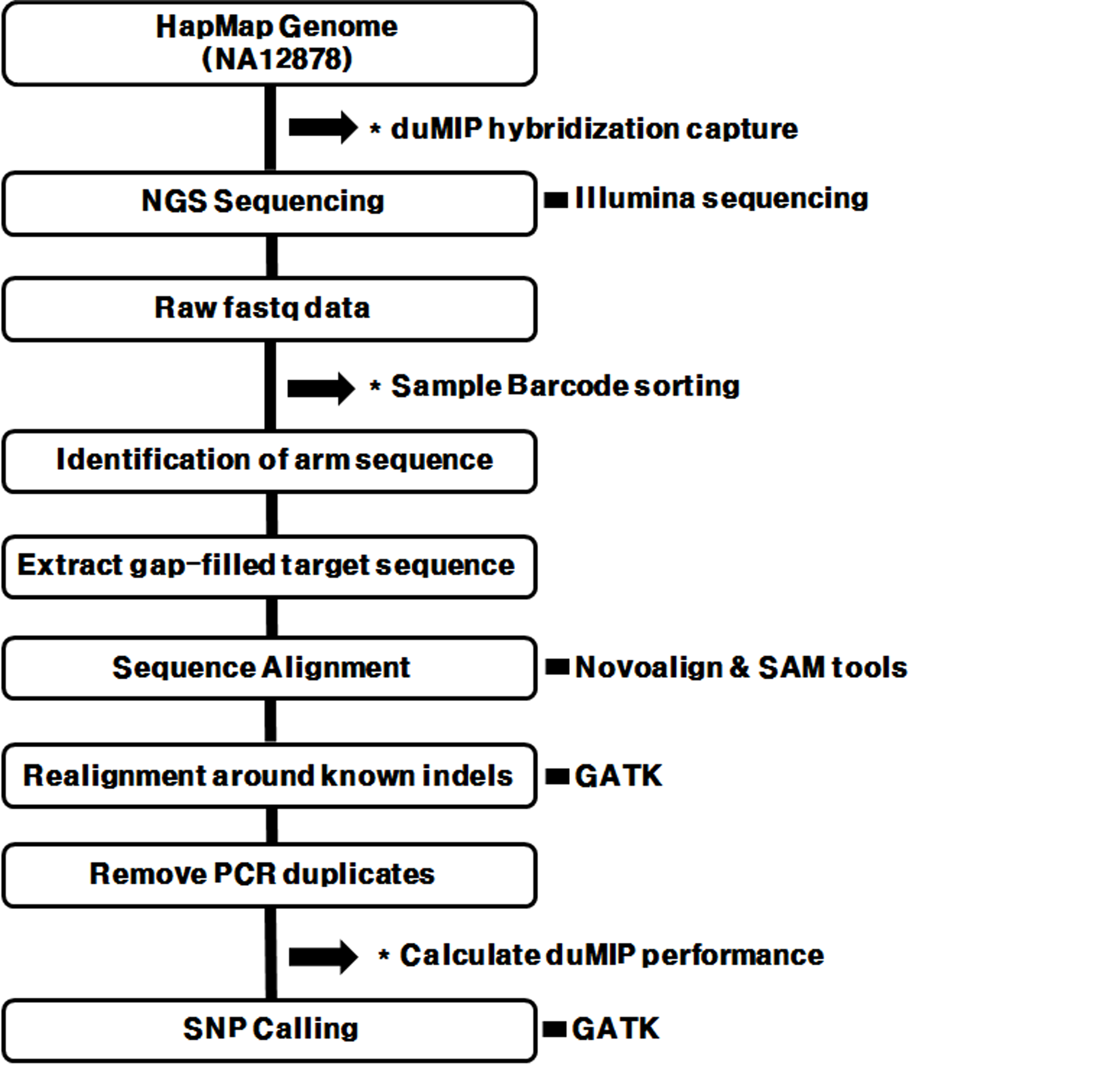


Supplementary Figure S3. Capture abundance heat map of 11,510 microDuMIP set according to melting temperature and GC contents of arms


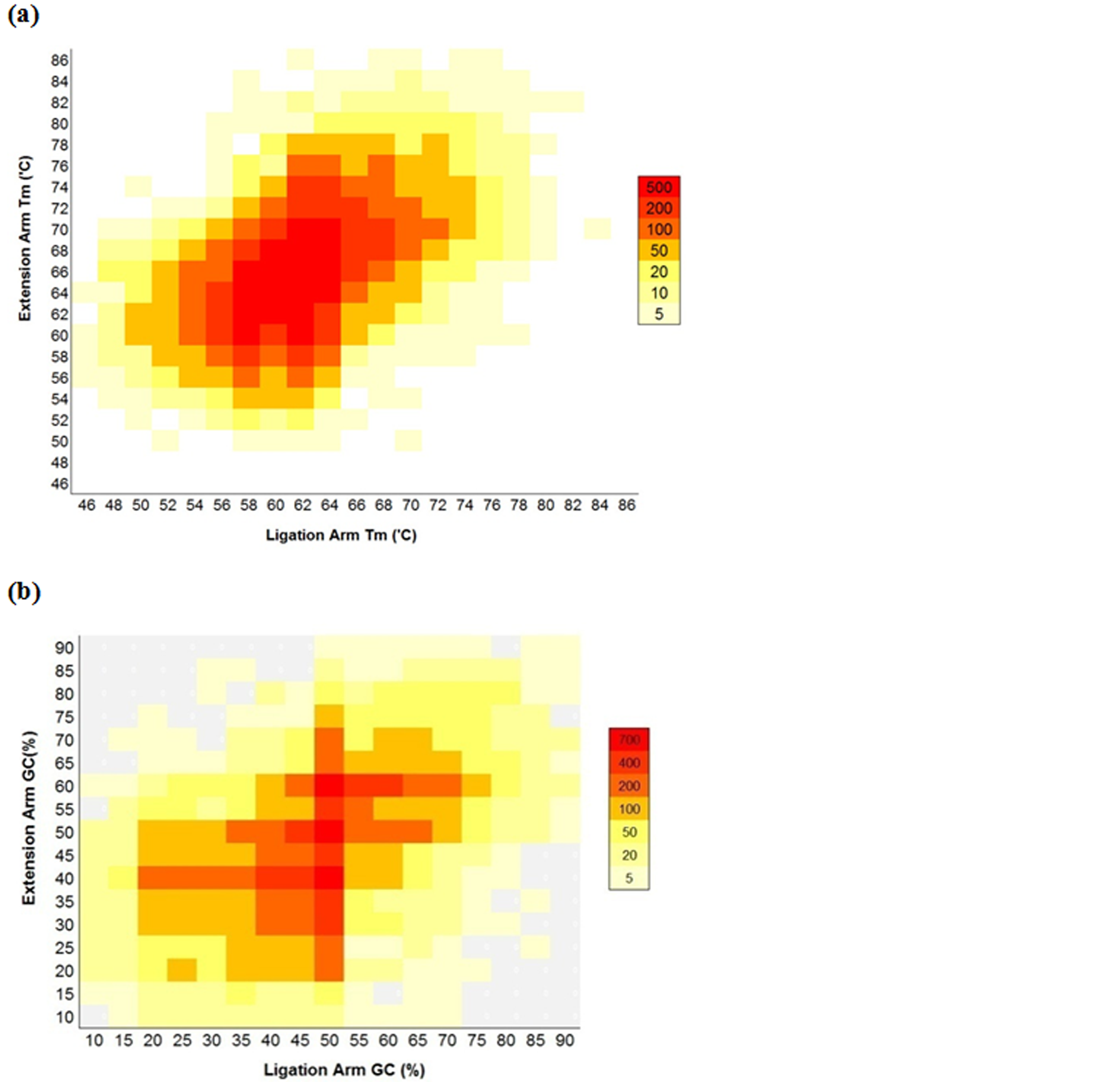


**(a)** The melting temperatures of ligation (x-axis) and extension arms (y-axis). The number in each bin is the average read depth from pairs of arms in 11,510 microDuMIPs used in SNP calling. The depths were highest when either the extension or ligation arm had an optimal temperature (60~70°C). Because microDuMIP capture used double-stranded capture probes, the extension arm sequence on the Watson strand acts as the ligation arm sequence on the Crick strand, and vice versa. Therefore, either arm having an optimal melting temperature can act as a ligation arm, resulting in higher depths. **(b)** GC contents (%) of the ligation (x-axis) and extension arms (y-axis). The depth distribution by GC contents is similar to that of melting temperatures, for the same reasons. The optimal GC content was 40~70%, but it is only one condition that affects read depth.

Supplementary Figure S4. Allele fractions before and after PCR duplicate filtering


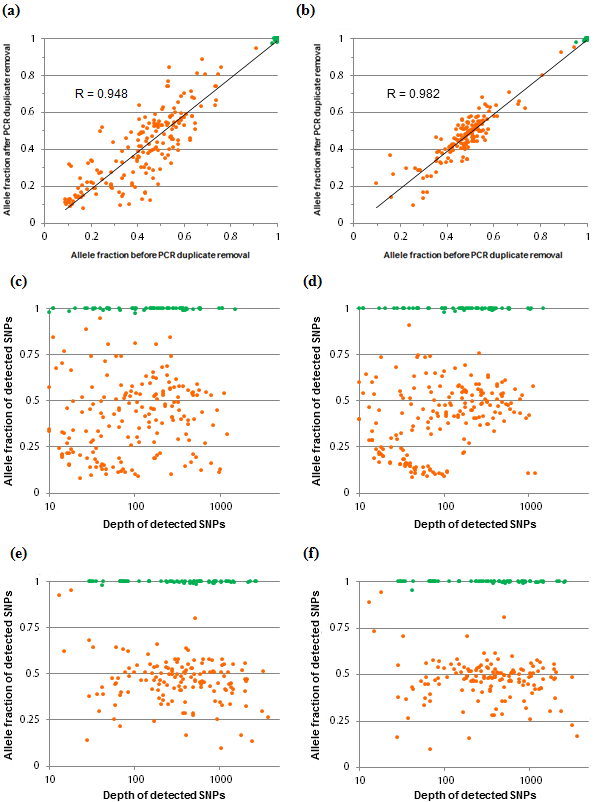


Correlation between allelic fractions of detected SNPs of NA12878 before (x-axis) and after (y-axis) removing PCR duplicates. (a) 50 ng of gDNA (Pearson coefficient *R*=0.948) (b) 500 ng of gDNA. (Pearson coefficient *R*=0.982) Allele fraction (y-axis) according to depth (x-axis) of detected SNPs with 50 ng of gDNA (c) before and (d) after removing PCR duplicates. Allele fraction according to depth with 500 ng of gDNA (e) before and (f) after removing PCR duplicates. Depths of detected SNPs after removing PCR duplicates (d, f) are the number of unique reads covering SNP loci. Homozygous SNPs (green) and heterozygous SNPs (orange) were called by GATK UnifiedGenotyper.

Supplementary Figure S5. Reproducibility of microDuMIP capture


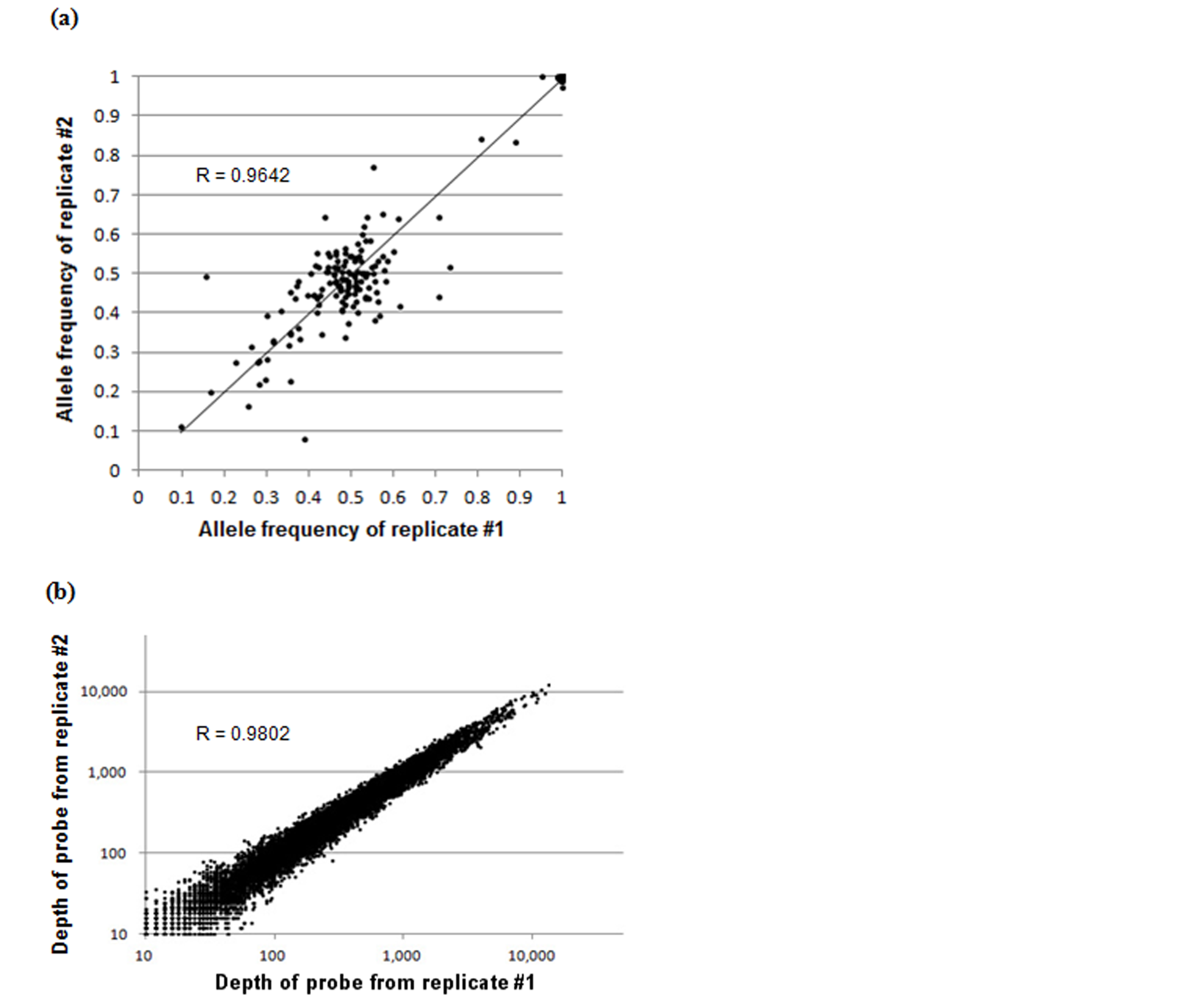


(a) Comparison of allele fractions of SNPs detected in two replicates with 500 ng of gDNA and 24 hr hybridization (Pearson coefficient *R*=0.9642) and (b) depths of microDuMIP (*R*=0.9802).

Supplementary Figure S6. Factors that affect the amount of circularized MIP product


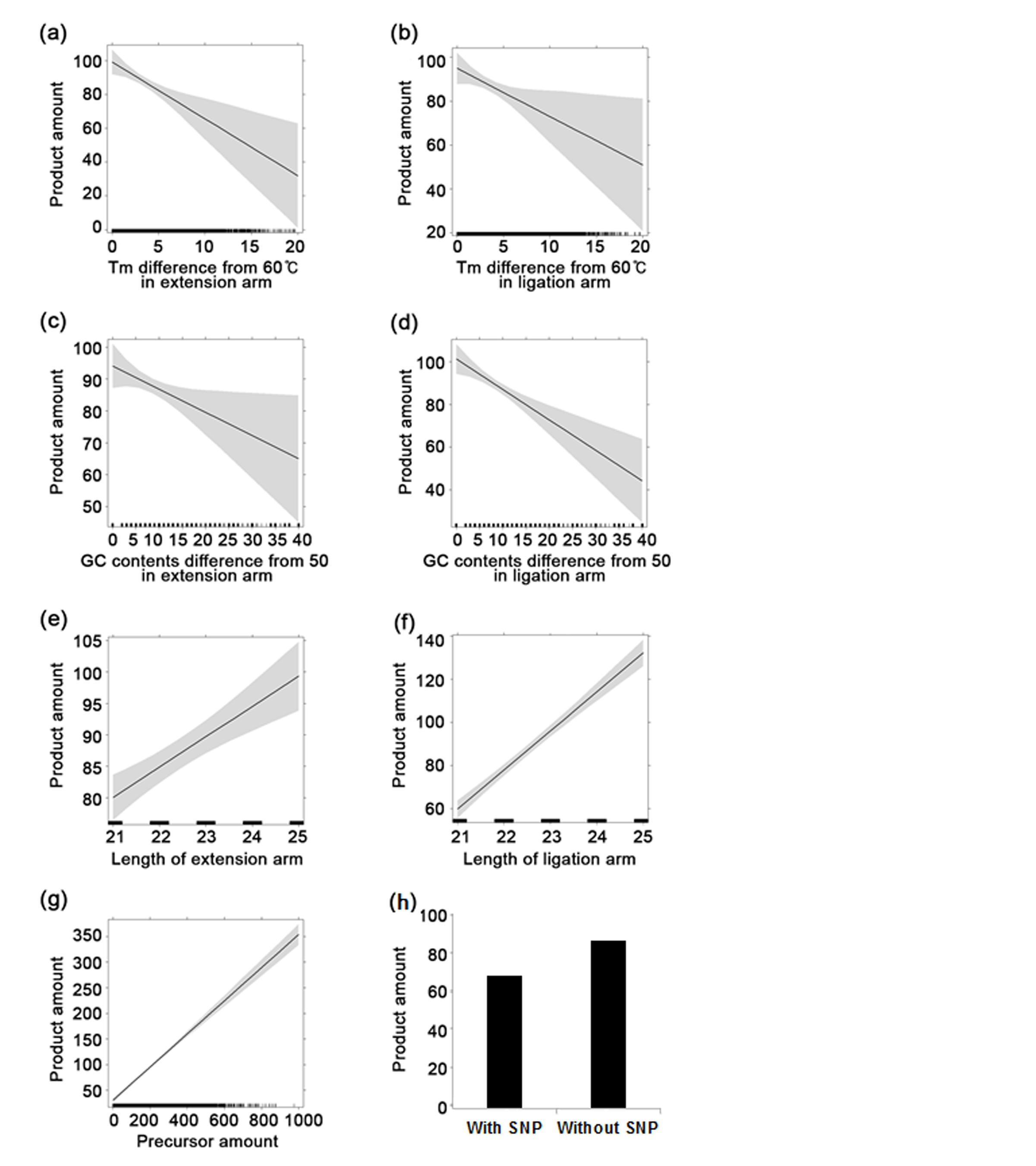


A linear model was applied and the shaded area represents the 95% confidence interval. Tm of the (a) extension and (b) ligation arms at temperatures other than 60°C were found to decrease the amount of circularized MIP product (*p*=0.00047 and *p*=0.019, respectively). (c) GC contents of the extension and (d) ligation arms other than 50% also decreased the amount of circularized MIP product (*p*=0.03 and *p*=1.62×10^-5^, respectively). (e) Extension arm length and (f) ligation arm length increased the amount of MIP product (*p*=7.23×10^-7^ and *p*<2×10^-16^, respectively). (g) Precursor amounts were highly correlated with the amount of circularized MIP product (*p*<2×10^-16^). (h) SNPs on arm sequences affected the amount of circularized MIP product (*p*<0.0013, *t*-test).

Supplementary Figure S7. The abundance of microDuMIP precursors


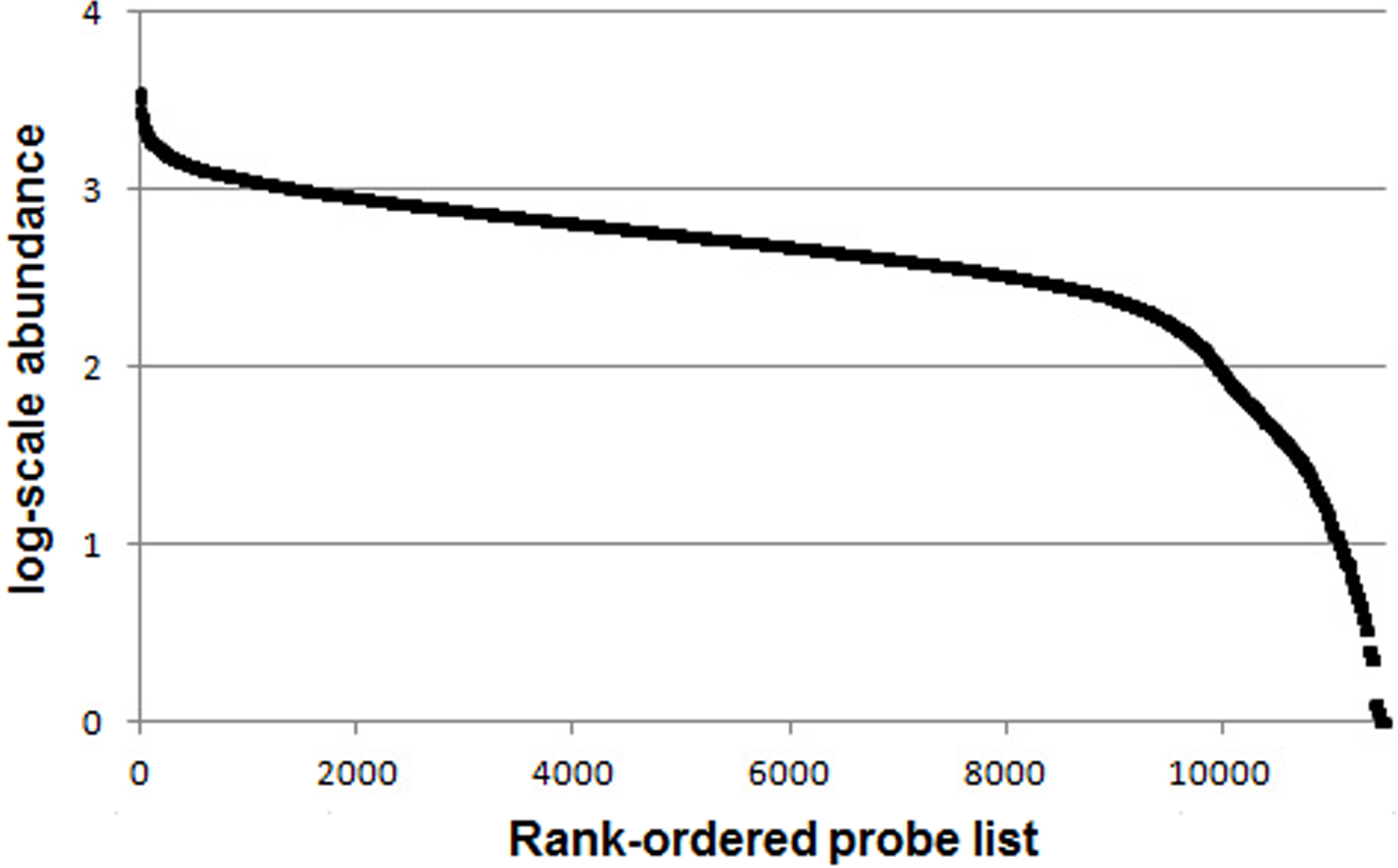


The abundance of microDuMIP precursor (y-axis) according to rank-ordered microDuMIP (x-axis) showed that the distribution of precursors was not fully uniform.

Supplementary Figure S8. Self-ligation products of probe during denaturation together with polymerization and ligation.


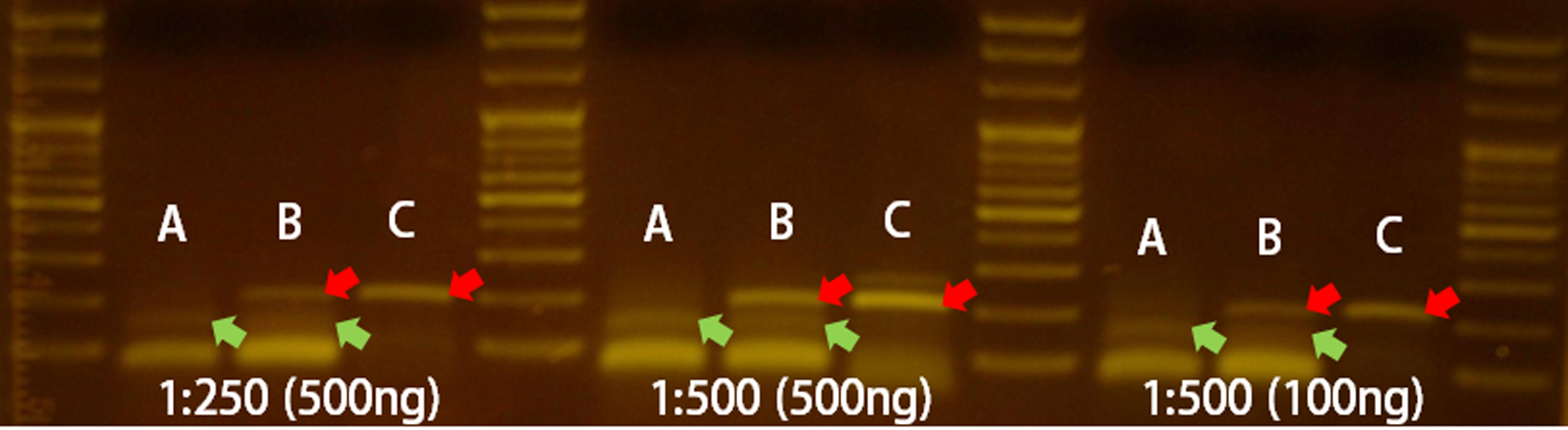


Since ligase and polymerase are heat stable proteins, the gap-filling reagents (i.e. ligase, polymerase, and dNTPs) may add to the reaction tubes before denaturation (O'Roak *et al*, Science 2012). We performed the capture using three sets of conditions: 1) 500 ng of gDNA with a 1:250 gDNA:probe ratio (left); 2) 500 ng of gDNA with a 1:500 gDNA:probe ratio (middle); or 3) 100 ng of gDNA with a 1:500 gDNA:probe ratio (right). All other conditions were held constant; that is, 10X dNTPs and a 24 hr hybridization time. Post-capture PCR amplified products were compared when adding reagents before denaturation **(B)** and after denaturation **(C)**. Control reaction tubes with only probes and reagents (no gDNA) were also prepared **(A)**. Both the desired product bands (red arrow) and self-ligation bands (green arrow) are marked.

In all conditions, self-ligation bands were observed in B (that is, when adding gap-filling reagents before denaturation) but not in C (adding reagents after denaturation). However, the bands were not dominant, were clearly distinguishable, and could be separated by gel-extraction. Further, the intensity of self-ligation bands decreased, as the amount of probe decreased. Capture conditions containing 100 ng gDNA and a 1:500 gDNA:probe ratio (right) are similar to those in the O'Roak study (<120 ng of gDNA with a 1:200 to 1:800 gDNA:probe ratio), and there was only a weak self-ligation band under these conditions, suggesting that gel purification would not be required. Therefore, adding the gap-filling reagents before denaturation can simplify this experimental step, with little probe self-ligation, especially when using a smaller amount of probes.

Supplementary Figure S9. The percentage of PCR duplicates by read depth


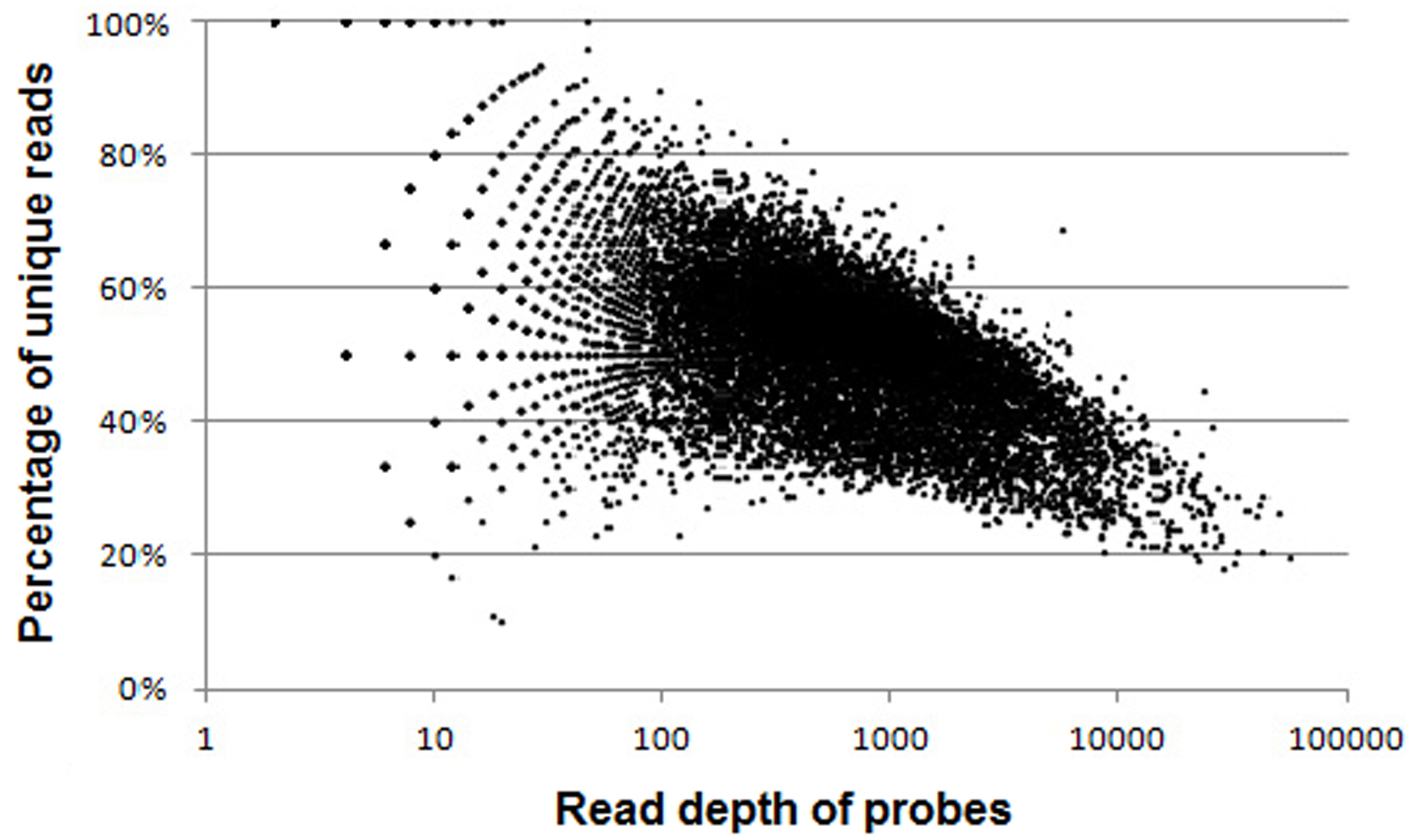


As read depth of target regions corresponding to probes increased, the number of PCR duplicates increased.
